# Altered Neuromodulatory Drive May Contribute to Exaggerated Tonic Vibration Reflexes in Chronic Hemiparetic Stroke

**DOI:** 10.3389/fnhum.2018.00131

**Published:** 2018-04-09

**Authors:** Jacob G. McPherson, Laura M. McPherson, Christopher K. Thompson, Michael D. Ellis, Charles J. Heckman, Julius P. A. Dewald

**Affiliations:** ^1^Department of Biomedical Engineering, Florida International University, Miami, FL, United States; ^2^Department of Physical Therapy and Human Movement Sciences, Northwestern University Feinberg School of Medicine, Chicago, IL, United States; ^3^Department of Physical Therapy, Florida International University, Miami, FL, United States; ^4^Department of Physical Therapy, Temple University, Philadelphia, PA, United States; ^5^Department of Physiology, Northwestern University Feinberg School of Medicine, Chicago, IL, United States; ^6^Department of Biomedical Engineering, Northwestern University, Evanston, IL, United States

**Keywords:** stroke, motor control, stretch reflex, bulbospinal monoaminergic drive, motoneurons, sensorimotor integration

## Abstract

Exaggerated stretch-sensitive reflexes are a common finding in elbow flexors of the contralesional arm in chronic hemiparetic stroke, particularly when muscles are not voluntarily activated prior to stretch. Previous investigations have suggested that this exaggeration could arise either from an abnormal tonic ionotropic drive to motoneuron pools innervating the paretic limbs, which could bring additional motor units near firing threshold, or from an increased influence of descending monoaminergic neuromodulatory pathways, which could depolarize motoneurons and amplify their responses to synaptic inputs. However, previous investigations have been unable to differentiate between these explanations, leaving the source(s) of this excitability increase unclear. Here, we used tonic vibration reflexes (TVRs) during voluntary muscle contractions of increasing magnitude to infer the sources of spinal motor excitability in individuals with chronic hemiparetic stroke. We show that when the paretic and non-paretic elbow flexors are preactivated to the same percentage of maximum prior to vibration, TVRs remain significantly elevated in the paretic arm. We also show that the rate of vibration-induced torque development increases as a function of increasing preactivation in the paretic limb, even though the amplitude of vibration-induced torque remains conspicuously unchanged as preactivation increases. It is highly unlikely that these findings could be explained by a source that is either purely ionotropic or purely neuromodulatory, because matching preactivation should control for the effects of a potential ionotropic drive (and lead to comparable tonic vibration reflex responses between limbs), while a purely monoaminergic mechanism would increase reflex magnitude as a function of preactivation. Thus, our results suggest that increased excitability of motor pools innervating the paretic limb post-stroke is likely to arise from both ionotropic and neuromodulatory mechanisms.

## Introduction

Focal ischemic stroke causes changes in descending neural drive that alter spinal motor excitability (Stinear et al., 2007; Heckman et al., 2009; Bradnam et al., 2012; McMorland et al., 2015; McPherson et al., 2017, 2018; Owen et al., 2017). This can occur through direct effects on spinal neurons and/or by facilitating adaptive processes associated with shifts in the overall balance of spinal excitation and inhibition. In the chronic state post-stroke, spinal motor excitability generally increases in regions that control the paretic limbs; in regions innervating the non-paretic limbs, excitability remains at approximately pre-injury levels or is increased to a lesser degree (Lee et al., 1987; Powers et al., 1989; Thilmann et al., 1990, 1991; Ibrahim et al., 1993; McPherson et al., 2011, 2017; Hu et al., 2015). Increased spinal motor excitability could theoretically be related to alterations in ionotropic drive (i.e., signaling via ligand-gated channels that directly elicits excitatory or inhibitory post-synaptic potentials), neuromodulatory drive (i.e., signaling via metabotropic receptors to modify the intrinsic excitability of the cell), or both. However, it is currently unknown which of these options primarily underlies increased spinal motor excitability in paretic motor pools because the results of previous investigations could have plausibly been explained by either phenomenon alone (Powers et al., 1989; McPherson et al., 2008; Mottram et al., 2009, 2010).

Stretch-sensitive reflexes are a useful assay of spinal motor excitability. For example, exaggerated mechanical and electromyographical responses to imposed joint excursions in paretic limbs have proven to be both conspicuous and enduring findings in chronic hemiparetic stroke. These reflex exaggerations are known clinically as spasticity (Lance, 1980). Reflexive contractions developed during maintained muscle or tendon vibration can also be used to assess spinal motor excitability (Eklund and Hagbarth, 1966; Hagbarth and Eklund, 1966; Gillies et al., 1971; Matthews, 1984; McPherson et al., 2008). These contractions are known as tonic vibration reflexes (TVRs). We have previously shown that TVRs are dramatically amplified in resting muscles of the paretic arm compared to homologous muscles of the non-paretic arm, and robust muscle activation persists in the paretic arm long after removal of the vibration (McPherson et al., 2008). The finding of both amplification and prolongation of motor output in paretic muscles is important because it may point to an increased influence of descending monoaminergic neuromodulatory pathways. Indeed, a hallmark of monoaminergic actions on somatic motoneurons is the development of persistent inward currents (PICs) in motoneuron dendrites, which uniquely amplify and prolong motoneuron output in response to excitatory synaptic inputs (Schwindt and Crill, 1977; Powers and Binder, 2001; Heckman et al., 2003, 2009).

However, some signs of an increased neuromodulatory influence appear to diminish when muscles are volitionally preactivated. For example, we and others have demonstrated that stretch reflex amplitude equilibrates between paretic and non-paretic muscles when the muscles are preactivated to the same extent prior to stretch (Lee et al., 1987; Burne et al., 2005; Mottram et al., 2009, 2010; McPherson et al., 2017). Additionally, some estimates of PIC amplitude are indistinguishable between paretic and control muscles during volitional ramp contractions (Mottram et al., 2009, 2010). Although equivocal, these findings are usually taken as evidence that a tonic, low-level ionotropic drive mediates the resting excitability imbalance between arms rather than an increased neuromodulatory drive (Mottram et al., 2009, 2010). In this scenario, the tonic ionotropic drive provides a sub-threshold depolarization to the resting motoneuron pool. The pre-reflex volitional descending drive depolarizes the motoneuron pool above spiking threshold, eclipsing the tonic ionotropic drive and effectively washing out the resting excitability imbalance. As a result, reflex amplitude is comparable in both limbs and increases as a function of pre-reflex muscle activation.

Here, we characterize TVRs in elbow flexors of the paretic and non-paretic arms of individuals with chronic hemiparetic stroke at three levels of preactivation. In particular, we quantify TVR-evoked elbow flexion joint torque amplitude and rise time, metrics that can be used to infer the spinal neuromodulatory state (McPherson et al., 2008; Revill and Fuglevand, 2017). We predicted that TVR-evoked torque would equilibrate between paretic and non-paretic arms when muscles were preactivated to the same degree prior to vibration, based on parallels with torque responses to stretch reflexes elicited by imposed joint excursion. Contrary to our prediction, we found pronounced amplification of TVR responses in the paretic arm despite matching preactivation levels between limbs. Perhaps more conspicuously, however, the magnitude of TVR-evoked torque did not scale with preactivation level in either limb. This finding stands in contrast to the well-documented positive correlation between preactivation level and reflex amplitude in response to imposed joint excursion (Ibrahim et al., 1993; Burne et al., 2005; McPherson et al., 2017). We also found that the rise time of TVR-evoked torque was significantly more rapid with increasing preactivation in the paretic limb compared to the non-paretic limb. Given these surprising results, we then corroborated our findings in a proof-of-principle decerebrate cat preparation that also allowed visualization of individual motor unit firing characteristics. In this model, descending monoaminergic drive is elevated and invariant to synaptic input, and force output is *not* influenced by volition. Thus, force output in this model is due only to three factors: the sustained monoaminergic drive, Ia input (via vibration), and the resulting input-output function of the motoneurons. We interpret these results as evidence that both an ionotropic and monoaminergic neuromodulatory drive are likely to contribute to the apparent increased excitability of motor pools innervating paretic muscles post-stroke.

## Materials and methods

### Human-subjects experiments

#### Participant characteristics and ethics statement

Ten individuals fulfilled all criteria for involvement in the study and subsequently completed the experimental protocol. The same individuals also participated in our original study of TVR responses post-stroke (McPherson et al., 2008). Each of these individuals (mean age: 59 ± 10 yrs) sustained a first-ever cortical or subcortical stroke at least 1 year prior to enrollment in the investigation (range: 40–124 months) and had lingering motor deficits on one side of the body. Fugl-Meyer motor assessment (FMA) scores ranged from 13 to 43 of a possible 66, representing severe to moderate impairment, and Ashworth scores (available for 6 of 10 participants) ranged from 2 to 4 (scoring: 0, 1, 2, 3, 4). Participant demographic and clinical data are summarized in Table 1.

**Table 1:** Participant demographic and clinical data.

**Participant**	**Age**	**Biological sex**	**Paretic arm**	**Fugl-meyer motor assessment**	**Ashworth score**
1	68	M	R	18	2
2	40	M	L	40	2
3	53	F	R	13	2
4	59	M	R	14	4
5	61	M	L	43	2
6	50	F	R	21	2
7	65	M	L	18	NA
8	59	M	L	24	NA
9	77	M	R	30	NA
10	51	F	R	26	NA

*Fugl-Meyer: 0–66; Ashworth: 0, 1, 2, 3, 4; NA, not available*.

For inclusion in the study, all participants were required to possess at least 90 degrees passive range of motion in shoulder flexion, shoulder abduction, and elbow flexion/extension. Participants were additionally required to exhibit some volitional control of elbow flexion/extension in order to obtain maximum voluntary torques (MVTs). The absence of inflammatory conditions at the shoulder, elbow, wrist and fingers was verified by overpressure at the end-range of motion. Potential participants were excluded from the study if the demonstrated minimal (50–66 on the FMA) or very severe (0–9 on the FMA) impairment, significant impairment of upper extremity tactile sensation or proprioception (O'Sullivan and Schmitz, 2001), or difficulties sitting for extended periods of time. All participants were required to have discontinued use of antispastic medications at least 6 months prior to enrollment; more recent or ongoing use was grounds for exclusion from the study.

All participants provided informed consent to participate in the investigation, which was approved by the Institutional Review Board of Northwestern University in accordance with the ethical standards stipulated by the 1964 Declaration of Helsinki for research involving human participants.

#### Experimental setup

Participants were secured to a Biodex experimental chair (Biodex Medical Systems, Shirley, NY) by shoulder and lap belt restraints. The forearm, wrist and hand of each participant's paretic and non-paretic arms were fitted with custom fiberglass casts. The casted arm was coupled at the level of the wrist to a 6 degree-of-freedom load cell (Model 45E15A; JR3, Woodland, CA), and the limb was positioned such that it retained 75° of shoulder abduction, 40° of shoulder flexion, and 90° of elbow flexion. The test apparatus supported the weight of the limb throughout these experimental protocols.

#### Experimental protocol

While interfaced with the isometric testing setup described above, participants were first required to generate MVTs in elbow flexion and extension. Visual feedback of performance was provided in real time for all trials. To obtain a reliable estimate of the true maximum torque capability in each direction, collection of MVTs continued until 3 trials were obtained within 10% of one another, without the last trial being the greatest.

For the TVR protocol, participants remained interfaced with the isometric setup used for testing MVTs, and a therapeutic massage vibrator (frequency: 112 Hz, model 91, Daito-Thrive, Showa-cho, Japan) was placed over the distal muscle belly of the biceps brachii. TVR trial onset and offset were indicated to the participant and experimentalist by auditory cues. TVR trials began after the experimentalist determined that the participant was fully relaxed (via real-time EMG and torque feedback). Upon trial onset, participants were first instructed to generate and maintain either 5 or 15% of their elbow flexion MVT, guided by visual feedback. Once the requisite torque was achieved, visual feedback was extinguished, and vibration commenced. The vibratory stimulus lasted for 5 s. Following each trial, participants were given at least a 15–30 s rest period to allow the limb to relax before beginning the next trial. First-pass trial acceptability was examined on-line by visual feedback provided to the experimentalist, and trials were excluded from further analysis if postural or unconstrained extremity movements occurred during the trial or if participants closed their eyes and appeared to fall asleep. Approximately 20 TVR response trials were collected at each of the 5 and 15% elbow flexion levels, and the protocol was performed on both the paretic and non-paretic limbs.

#### Data analysis

Analyses of TVR-evoked elbow flexion torque were used as outcome measures in this study, computed using custom Matlab software (The MathWorks, Natick, MA). A Jacobian-based algorithm converted forces and moments recorded by the loadcell into elbow flexion/extension torques. The resulting torque curves were filtered with an eighth-order low-pass Butterworth filter with cutoff frequency of 50 Hz. Window averages of individual TVR trials were taken from 3.5 to 5.0 s after trial onset, corresponding to the preactivation phase, and from 9.5 to 10.0 s post-trial onset, corresponding to the last 500 ms of the vibration phase. We did not extract data during the post-vibration period because reflexive torque and EMG responses could not be reliably decoupled from volitional efforts to decrease elbow flexion torque. Preactivation windows and during-vibration windows were then ensemble-averaged for each participant and limb (paretic and non-paretic) for use in subsequent group analyses.

To quantify TVR-evoked torque amplitude, we computed the change in torque from preactivation to during-vibration for each participant's ensemble-averaged torque response. We computed this difference at each preactivation level (0, 5, 15%), and these values were subsequently used in group statistical analyses. Because of the likely difference in elbow flexion strength between paretic and non-paretic limbs, we calculated the TVR-evoked torque amplitude using both unnormalized torque (Nm) and normalized torque (% MVT). This dual approach ensured that between-limb results would not be inflated by normalization of paretic torque to lower MVT values.

We used two approaches to quantify the temporal profile of the rising phase of the TVR-evoked torque response. First, we computed the slope of a linear fit between each participant's preactivation torque and the time at which TVR-evoked torque reached 85% of the maximum stable response (Equation 1):

(1) $$\begin{array}{l} Rising\;slope\;of\;TVR\;evoked\;torque=\\ \frac{\left(torque_{85\text{\%}max}-torque_{preac}\right)}{time_{85\text{\%}max}-time_{Vib.ON}}\left[\%MVT\right]/sec \end{array}$$

Second, because TVR-evoked torque generally exhibits a non-linear temporal profile, we used non-linear least squares estimation to determine the optimal fit of each group mean ensemble-averaged torque response (i.e., paretic and non-paretic, each at 5 and 15% preactivation) to a function of the form:

(2) $$\begin{array}{l} Modeled\;TVR\;evoked\;torque=a\text{*}\left(1-e^{-t/b}\right) \end{array}$$

In this equation, term *a* generally represents the amplitude of the TVR-evoked torque (add preactivation torque to *a* to approximate the final steady-state torque amplitude in the plots below), term *b* is the time constant describing the rate of rise of TVR-evoked torque, and *t* is elapsed time. The model was fit to experimental data from the time of vibration onset to the time of vibration offset; *a* and *b* are coefficients fit by the optimization routine and *t* is elapsed time. The parameters of this model are not intended to reflect specific biophysical features of motoneuron firing.

#### Statistical analysis

For TVR-evoked torque amplitude, all statistical analyses were calculated on the normalized torque values. Unnormalized torque values are presented for comparison. A 2 × 3 repeated measures ANOVA was used to determine the main effects of limb (paretic, non-paretic) and preactivation level (0, 5, 15%), as well as the limb-by-preactivation level interaction, on normalized TVR-evoked torque amplitude. To reiterate, all participants included in this investigation were included in our original study of TVR responses post-stroke (McPherson et al., 2008), enabling data to be pooled across experiments to generate the new analyses presented here. Two sets of *post-hoc t*-tests were used. First, *t*-tests were used to determine differences between limbs at each preactivation level, with Bonferroni correction for multiple comparisons. Second, *t*-tests within each limb were used to evaluate differences between all combinations of preactivation levels, using Tukey correction for multiple comparisons within each limb.

For the slope of TVR-evoked torque, there were 60 total values (2 limbs × 3 preactivation levels × 10 participants). Several outliers were detected in both paretic and non-paretic values, which were excluded from further analysis. Outliers were defined as values exceeding 3 scaled median absolute deviations from the median. Given that a 2-way repeated measures ANOVA (which is most appropriate for this experimental design) requires no missing cases, the following method was used to account for the missing data and allow for a repeated measures ANOVA. First, if a participant had missing data for more than one preactivation level, all data for that participant was removed. This resulted in removing data from two participants for the non-paretic limb (with *N* = 8 remaining) and data from one participant in the paretic limb (with *N* = 9 remaining). Together, these removals reduced the total number of torque values from 60 to 51. There were two missing cases at one preactivation level for the non-paretic limb (for one participant at 0% and for another participant at 15%). Values for these cases were imputed by taking the average of values from the seven other participants. For the paretic limb, there was one missing case at the 0% condition, and the value was imputed by taking the average of the eight other participants for this condition. As a result, the number of total values that were imputed was 3 out of 51, or 5.9%. This method allowed for an ANOVA with a repeated factor of preactivation; however, because the number of participants for each limb was not the same, the factor of limb could not be repeated. Therefore, a mixed-model 2 × 3 ANOVA was used to determine the main effects of limb (paretic, non-paretic) and preactivation level (repeated measure; 0, 5, 15%) as well as the limb-by-preactivation level interaction on TVR-evoked slope magnitude. Between-limb *post-hoc t*-tests were used to determine differences in slope between paretic and non-paretic limbs at each preactivation level, with Bonferroni correction for multiple comparisons. Within-limb *post-hoc t*-tests were used to evaluate differences between all combinations of preactivation levels, using Tukey correction for multiple comparisons within each limb. Finally, a 2-way non-repeated measures ANOVA was also calculated without accounting for the missing data to ensure that the above analyses were not biased by methods used for accounting for missing data. Results from this ANOVA were virtually identical to those from the 2-way repeated measures ANOVA that is presented in the Results section below, with the same significant ANOVA effects and *post-hoc* test results.

To evaluate the goodness-of-fit of the non-linear model of TVR-evoked torque, we computed the variance accounted for (VAF) and sum-of-squared error (SSE) of the model. The effect of preactivation on the model parameters associated with the TVR-evoked portion of the torque profile was examined qualitatively for each limb.

Results of all analyses were considered significant at the *p* < 0.05 level, and *p*-values for the *t*-tests are presented in the results after application of corrections for multiple comparisons. All statistical analyses were performed in Prism (GraphPad software, Inc., version 7.0a).

### Animal experiments

As a first step toward providing additional mechanistic context for our human-subjects findings, we conducted a set of proof-of-principle TVR experiments in a decerebrate cat model. The primary goal of this model was to determine the impact of increasing preactivation level on the amplitude and rate of rise of TVR-evoked force. We chose the decerebrate cat preparation because it has a well-documented elevation of descending monoaminergic drive that does not scale with motor output (Crone et al., 1988) thus paralleling one potential mechanism underlying our chronic hemiparetic stroke findings. Further, we examined the motor unit firing patterns underlying the TVR-evoked forces to evaluate the effect of preactivation on motor unit rate modulation and recruitment during and after vibration.

#### Experimental setting and ethics statement

Data were also collected in one adult cat sourced from a designated breeding establishment for scientific research. The animal was housed at Northwestern University's Center for Comparative Medicine, an AAALAC accredited animal research program. All procedures were approved by the Institutional Animal Care and Use Committee at Northwestern University and conform with accepted ethics standards (Grundy, 2015).

#### Surgical procedure

Anesthesia was induced with 4% isoflurane and a 1:3 mixture of N_2_O and O_2_. Anesthetic depth was monitored via blood pressure, heart and respiratory rate, and withdrawal reflexes. Once surgically anesthetized, a tracheostomy was performed and a permanent tracheal tube was implanted. Isoflurane (0.5–2.5%) and gasses were delivered through the tube for the duration of the surgery. The animal was then transferred to a stereotaxic frame and immobilized by a head clamp, spinal clamp on the L2 dorsal vertebral process, and bilateral hip pins at the iliac crest. The left hindlimb was fixed with pins at the knee and clamps at the ankle, and the right hindlimb was secured using a clamp on the lower leg. The left soleus was dissected, isolated, and its distal tendon was attached to a load cell via a calcaneus bone chip in series with a linear variable differential transformer and customized voice coil. A distal, cutaneous branch of the right superficial peroneal nerve was dissected and a cuff electrode was secured around the nerve. The animal was then decerebrated at the precollicular level and anesthesia was discontinued (animals lack sentience after decerebration; Silverman et al., 2005). A thermistor was then placed in the esophagus and core temperature was maintained at 35–37°C. At the end of the experiment, the animal was euthanized using a 2 mM/kg solution of KCl in addition to a bilateral thoracotomy.

#### Data collection

Referenced monopolar EMG activity was acquired using a custom 64-channel electrode array that covered the surface of the exposed soleus. The array consisted of 64 individual rigid silver pins, 7.5 mm in length and 0.7 mm in diameter, configured in a 5 × 13 matrix with an interelectrode distance of 2.54 mm. A ground electrode was place on the animal's back and a reference electrode was placed on the upper thigh. EMG data were bandpass filtered (100–900 Hz), amplified (0.5–2 k) and digitized (5,120 Hz) by a 12-bit A/D converter (EMG-USB 2, 256-channel EMG amplifier, OT Bioelettronica, Torino, Italy). Force data from the soleus muscle were simultaneously acquired.

With the animal's hindlimbs fixed, the soleus was activated by stimulating the contralateral superficial peroneal nerve through a cuff electrode (voltage-controlled stimulation; 50 Hz; 1 ms pulse width). This elicits the crossed-extension reflex and a maintained contraction. The magnitude of contraction is generally proportional to the applied voltage, and here, we used voltages ranging from 3.5 to 5 V. This evoked sub-maximal contractions up to ~25% of maximum force output, as confirmed by the appearance of force saturation with voltages at or above 6 V in this animal. After at least 2 s of stimulation (to acquire a stable baseline preactivation), 3–5 s of vibration of the soleus tendon commenced (~130 Hz; ~80 μm). This elicited a TVR. Electrical stimulation of the contralateral peroneal nerve continued for the duration of vibration, and we varied the applied voltage between trials to explore the impact of vibration at a range of preactivation levels.

#### Data and statistical analysis

Force data from the soleus was analyzed similarly to the elbow flexion torque data from the human subjects experiments. Preactivation force was calculated as the average of force values generated during the 0.5 s prior to vibration onset. TVR-evoked force amplitude was calculated as the change in force from the preactivation value to the average torque between 1 and 2 s post-vibration onset. The rising slope of the TVR-evoked force was calculated as with Eqn. 1 for the human data. The time to 85% max TVR-evoked force was calculated relative to vibration onset. Separate Pearson correlation calculations were used to determine if increasing preactivation level was associated with changes in TVR-evoked force amplitude, rising slope of the TVR-evoked force, or the time to 85% max TVR-evoked force.

To examine motor unit recruitment and rate modulation patterns underlying the TVR-evoked force due to different levels of preactivation, multi-channel EMG data from each trial were decomposed into individual motor unit spike trains using a blind source separation approach (Holobar et al., 2010, 2014; Negro et al., 2016). The instantaneous firing rate for each motor unit spike train was calculated as the reciprocal of the inter-spike interval.

## Results

### TVR-evoked torque amplitude

**Figure 1:**
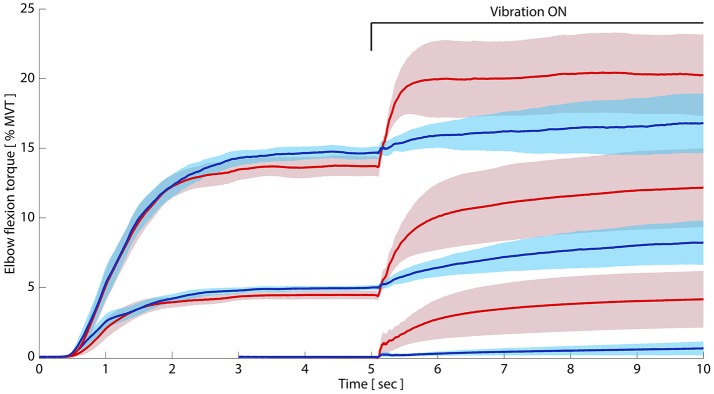
Paretic limb tonic vibration reflexes remain exaggerated despite preactivation. Paretic and non-paretic elbow flexors were preactivated to 5 and 15% of MVT, respectively, before vibration began. Despite matching preactivation levels between limbs, group average (*N* = 10) paretic TVRs (pink) significantly exceeded non-paretic TVRs (blue). Bottom paretic and non-paretic traces are TVR responses in relaxed elbow flexors, with data adapted with permission from McPherson et al. (2018). Shaded region around each TVR curve represents ± 95% confidence interval. *Y-axis:* elbow flexion torque as percent of maximum; *x-axis*: time in seconds. Permission to adapt images from McPherson et al. (2008) is afforded by the American Physiological Society under original author rights.

Across participants, maximum voluntary elbow flexion torque for the paretic limb averaged 34.85 Nm, whereas the non-paretic elbow flexion MVT averaged 62.29 Nm. Elicitation of the TVR in the paretic and non-paretic limbs in all cases resulted in an increase in net elbow flexion torque above the target preactivation level (Figure 1). Qualitatively, this torque developed rapidly upon vibration onset in the paretic limb and either monotonically increased during vibration or reached a stable plateau following the initial rising phase. The non-paretic limb demonstrated a more gradual increase in torque following vibration onset, which was also characterized by a monotonic rise or an initial increase followed by a plateau.

Table 2 shows the TVR-evoked torque amplitude (unnormalized and normalized; left and middle panels, respectively) for both limbs at the three preactivation levels, as well as the normalized absolute torque values during vibration (i.e., those including preactivation torque; right panel). The data from the 0% MVT level is reproduced with permission from our previous work (McPherson et al., 2008). At the 5% MVT preactivation level in the paretic limb, vibration induced an increase in torque from 4.23% MVT during the preactivation period to 12.02% MVT during vibration (difference: 7.79% MVT, 2.52 Nm). In the non-paretic limb, preactivation to 5% MVT led to a TVR-evoked torque increase from 4.92 to 8.15% MVT (difference: 3.23% MVT, 1.76 Nm; Table 2). At the 15% MVT preactivation level, vibration induced an increase in torque from 13.39 to 21.00% MVT in the paretic limb (difference: 7.41% MVT, 2.25 Nm) and an increase in torque from 14.46 to 16.79% MVT in the non-paretic limb (difference: 2.15% MVT, 0.88 Nm).

**Table 2:** TVR-evoked torque amplitude.

	**TVR-evoked torque amplitude (Difference from preactivation to vibration-ON)**						**Torque amplitude during vibration (incl. preactivation torque) (% MVT)**		
	**Unnormalized Torque (Nm)**			**Normalized Torque (%MVT)**					
**Preactivation**	**0%**	**5%**	**15%**	**0%**	**5%**	**15%**	**0%**	**5%**	**15%**
Paretic	1.20 ± 0.98	2.52 ± 1.32	2.25 ± 1.23%	3.75 ± 3.18%	7.79 ± 5.19%	7.41 ± 5.22%	4.48 ± 3.88%	12.02 ± 5.00%	21.00 ± 4.65%
Non-Paretic	0.35 ± 0.54	1.76 ± 1.27	0.88 ± 1.72	0.64 ± 0.94%	3.23 ± 2.55%	2.15 ± 3.52%	0.72 ± 1.03%	8.15 ± 2.59%	16.79 ± 3.65%

*Data presented as mean ± standard deviation*.

The 2 × 3 repeated measures ANOVA revealed significant main effects of limb (*p* = 0.004) and preactivation level (*p* = 0.0004) on normalized TVR-evoked torque amplitude, and a non-significant limb-by-preactivation level interaction (*p* = 0.16). Examining the significant main effect of limb, paretic limb values were greater than non-paretic values, averaging 6.32% MVT across pre-activation levels vs. 2.00% MVT. The uniform increase in paretic vs. non-paretic TVR-evoked torque amplitude at all preactivation levels (i.e., significant main effect of limb and non-significant limb-by-preactivation interaction) was contrary to our prediction that the torque response would become similar across limbs with preactivation vs. the relaxed condition.

Results from within-limb *post-hoc t*-tests are summarized in Table 3, top panel. Paretic normalized TVR-evoked torque amplitude values from the 0% condition were significantly less than those from the 5 and 15% conditions (*p* = 0.009 and *p* = 0.025, respectively). Notably, however, there was no difference in TVR-evoked torque amplitude in the paretic limb between trials with torque preactivation of 5 and 15% MVT (*p* = 0.84). The same analysis in the non-paretic limb revealed a significant difference between the 0 and 5% conditions only (*p* = 0.007); comparisons of 0 vs. 15% (*p* = 0.32) and 5 vs. 15% (*p* = 0.30) were not statistically significant. Results from between-limb *post-hoc t*-tests are summarized in Table 3, bottom panel. As expected from the significant main effect of limb and non-significant limb-by-preactivation interaction, paretic TVR-evoked torque amplitude values were higher than non-paretic values at all preactivation levels.

**Table 3:** *Post-hoc* comparisons of TVR-evoked torque.

	**TVR-evoked torque amplitude**		**Slope of torque rise**	
***Within-limb***	**Paretic**	**Non-paretic**	**Paretic**	**Non-paretic**
0% vs. 5% MVT	^**^	^**^	*ns*	*ns*
0% vs. 15% MVT	^*^	*ns*	^****^	*ns*
5% vs. 15% MVT	*ns*	*ns*	^****^	*ns*
***Between-limb***	**Paretic vs. Non-Paretic**		**Paretic vs. Non-Paretic**	
0% MVT	^**^		*ns*	
5% MVT	^****^		*ns*	
15% MVT	^****^		^****^	

Asterisks indicate significant differences at

* *p < 0.05*,

** p < 0.01, or

**** *p < 0.0001*.

*ns indicates p > 0.05. Post-hoc comparisons computed from normalized torque*.

### Slope of TVR-evoked torque

**Figure 2:**
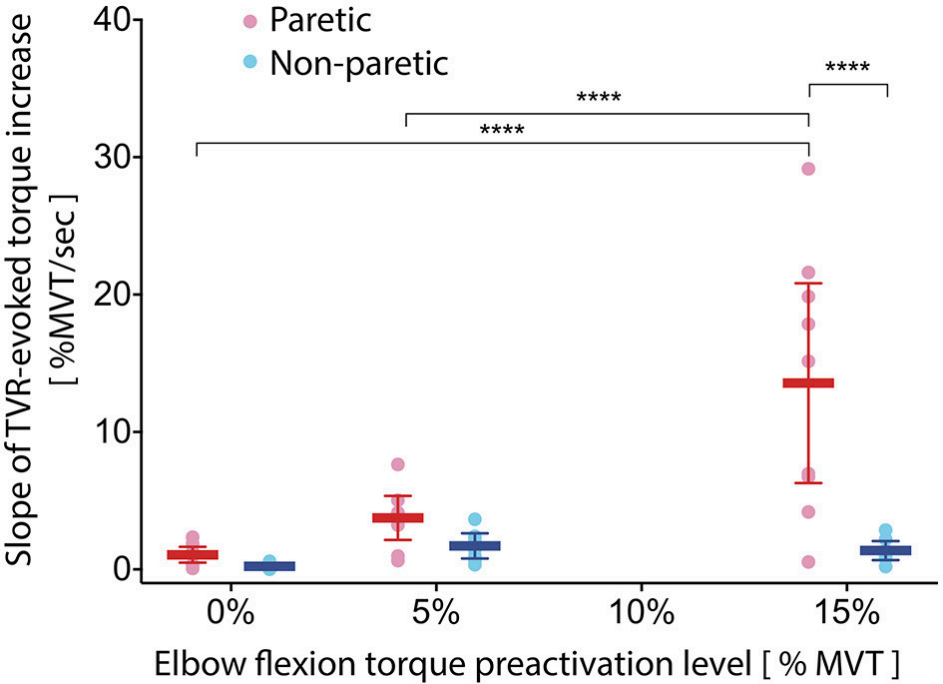
Slope of TVR-evoked torque increases with preactivation only in paretic limb. As preactivation level increases, the slope of TVR-evoked torque increases in the paretic (pink/red) but not the non-paretic (light blue/royal blue) elbow flexors. Differences between limbs emerge at the 15% preactivation level. Individual circles represent single participant values; horizontal lines indicate group means, with error bars corresponding to 95% confidence intervals. *Y-axis*: slope of TVR-evoked torque (%MVT per second); *x-axis*: elbow flexion torque preactivation level expressed as percent of MVT. ^****^p < 0.0001.

Qualitatively, the rate of rise of TVR-evoked torque in the paretic limb appears to increase with increasing preactivation. Further, the rate of rise in the paretic limb appears to be more rapid than that of the non-paretic limb at matched levels of preactivation (Figure 1). We evaluated these observations quantitatively by computing the slope of the rising TVR-evoked torque according to Equation (1), including a new analysis of TVR responses previously collected with 0% preactivation (adapted from McPherson et al., 2008; Figure 2). The 2 × 3 mixed-model ANOVA revealed significant main effects of limb (*p* = 0.002) and preactivation level (*p* < 0.0001) as well as a significant limb-by-preactivation level interaction (*p* = 0.0002). A 2-way non-repeated measures ANOVA, without imputation, likewise revealed significant main effects of limb (*p* = 0.0003) and preactivation level (*p* = 0.0002) and a significant limb-by-preactivation level interaction (*p* = 0.0008). The nature of the significant interaction can be appreciated from visual inspection of Figure 2. Slope values for the paretic limb increased with preactivation level, but slope values for the non-paretic limb remained constant. Paretic group mean slope of TVR-evoked torque averaged 1.0, 3.5, and 12.5% MVT/s (for 0, 5, and 15% MVT preactivation levels, respectively), and 0.2, 1.6, and 1.4% MVT/s in the non-paretic limb. As such, the significant main effect of preactivation level was driven by the paretic limb values.

*Post-hoc t*-tests comparing between-limb differences in the slope of TVR-evoked torque at each pre-activation level revealed a significant difference at the 15% condition (*p* < 0.0001) and not the 0 or 5% conditions (*p* = 0.99 and *p* = 0.95, respectively) (Table 3, bottom panel).

*Post-hoc t*-tests comparing within-limb differences in the slope of TVR-evoked torque between each combination of the three preactivation levels (Table 3, top panel) revealed that in the paretic limb, values for the 0 and 5% conditions were significantly less than those of the 15% condition (*p* < 0.0001 for both comparisons), but there was no significant difference between values for the 0 and 5% conditions (*p* = 0.31). The same analysis in the non-paretic limb revealed no significant differences in the slope of TVR-evoked torque among any preactivation level comparisons (*p*-values ranging from 0.72 to 0.98).

### Modeled TVR-evoked torque profile

**Figure 3:**
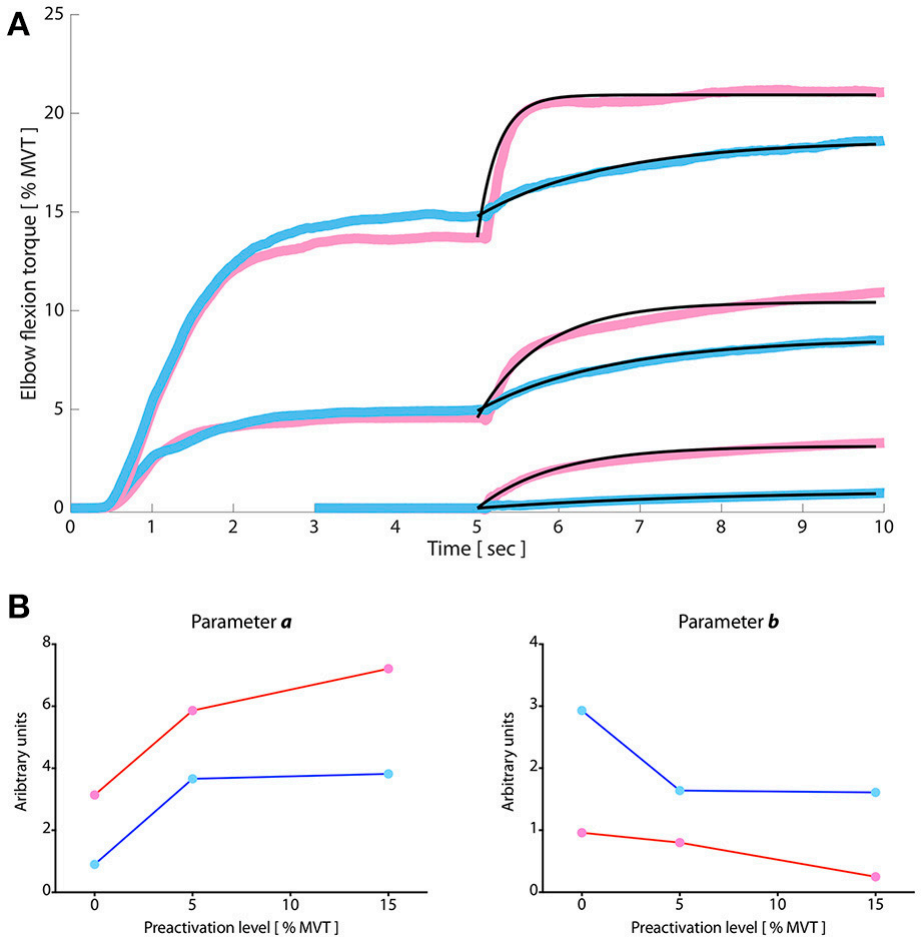
TVR torque responses modeled by a composition of exponential functions. **(A)** Paretic and non-paretic TVR responses were well fit by exponential functions (black lines from 5 to 10 s), with Variances Accounted For of approximately >94% in all cases. Vibration commences at 5 s and continues for the duration of the plot. *Y-axis:* elbow flexion torque, expressed as a percentage of max; *x-axis:* time in seconds. Pink (paretic) and blue (non-paretic) traces are group averaged data (*N* = 10). **(B)**. Optimal parameters of exponential fit expressed as function of preactivation level. Pink/red: paretic limb; blue: non-paretic limb. Qualitatively, parameter *a* is greater in the paretic than the non-paretic limb; it trends toward an increased magnitude with increasing preactivation in the paretic limb but plateaus in the non-paretic limb. Parameter *b* is lower in the paretic limb than the non-paretic limb and decreases as a function of increasing preactivation. Permission to adapt images from McPherson et al. (2008) is afforded by the American Physiological Society under original author rights.

Because the temporal profile of TVR-evoked torque is non-linear, particularly during the first ~1 s of vibration, we also modeled TVR-evoked torque as an exponential function according to Equation (2). All data were well-fit by this model, as can be appreciated visually in Figure 3A (black lines superimposed on data records). In Table 4, we present the optimal parameters for each group mean torque response at each preactivation level, as well as the SSE and VAF for each fit.

**Table 4:** Modeled TVR-evoked torque.

	**Preactivation level (% MVT)**	***a***	***b***	**SSE**	**VAF**
Paretic	0%	3.14	0.96	97.3	97.14%
	5%	5.86	0.8	549.37	94.21%
	15%	7.21	0.25	247.17	96.33%
Non-paretic	0%	0.9	2.93	2.98	98.90%
	5%	3.66	1.64	31.84	99.44%
	15%	3.82	1.61	111.9	98.13%

In Figure 3B, we plot parameters *a* and *b* from the model (left and right panels, respectively) for the paretic (pink) and non-paretic (blue) limbs as a function of preactivation level. These two parameters describe the shape of the during-vibration profile. Parameter *a*, most closely associated with the amplitude of TVR-evoked torque, tends to increase as a function of preactivation level for the paretic limb but not the non-paretic limb. Parameter *b*, reflective of the rate-of-rise of the TVR-evoked torque, is lower overall in the paretic limb—indicating more rapid torque development during vibration—and qualitatively appears to decrease as preactivation level increases.

**Figure 4:**
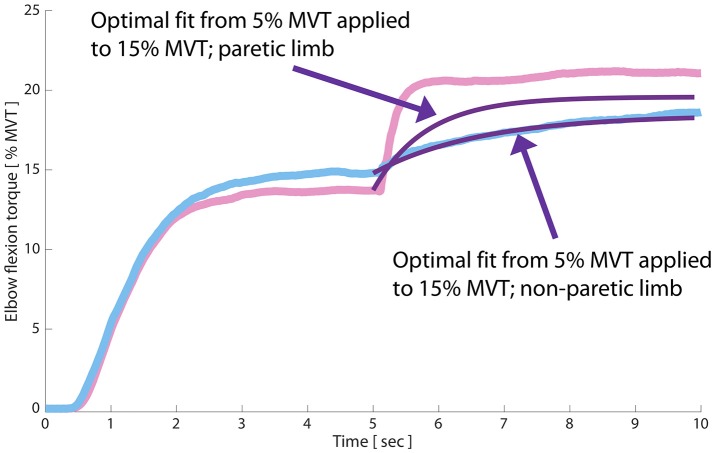
Initial TVR-evoked torque development is invariant to preactivation magnitude in the non-paretic but not the paretic limb. Blue trace: non-paretic limb at 15% MVT preactivation; pink trace: paretic limb; black traces: optimal fits of an exponential composition to paretic and non-paretic data, respectively; purple traces: overlay of optimal fit of each limb's TVR-evoked torque at 5% MVT preactivation onto the 15% MVT preactivation records. In the non-paretic limb, the optimal fit at 5% MVT is able to explain ~98% of the variance in the torque response at 15% MVT preactivation. Conversely, the 5% MVT fit is only able to explain ~75% of the variance in torque response at 15% MVT in the paretic limb.

Because parameters of modeled TVR-evoked torque data from the non-paretic limb had relatively less change across preactivation levels than those of the paretic limb, we also computed the VAF when applying the model fit *from* the 5% preactivation data *to* the 15% preactivation data (separately for the non-paretic and paretic limbs). This manipulation gives an estimate of similarity between TVR-evoked responses at increasing preactivation levels, particularly during the rising phase of the contraction. We found that the optimal parameters for describing the 5% preactivation response in the non-paretic limb also accounted for 98.25% of the variability in the 15% preactivation trials in the same limb (Figure 4; purple trace overlaid on raw data). Conversely, for the paretic limb only 75.41% of the variability in the 15% preactivation data could be explained by the optimal fit to the 5% preactivation torque responses (down from 96.33%; Figure 4; purple trace overlaid on raw data). This finding, too, is consistent both with the relative invariance of linear slope in the non-paretic limb to preactivation level and the strong dependency of paretic limb linear slope on preactivation.

### TVR responses in decerebrate cat preparation

To examine mechanisms of why the TVR did not vary with preactivation level, we undertook studies of the TVR in a decerebrate cat preparation. We reasoned that if the same pre-activation invariance occurred in this preparation, it would reflect the basic input-output behavior of the motor pool in response to stable, sustained Ia input (in the presence of increased spinal monoamines) instead of some type of compensation by descending inputs.

**Figure 5:**
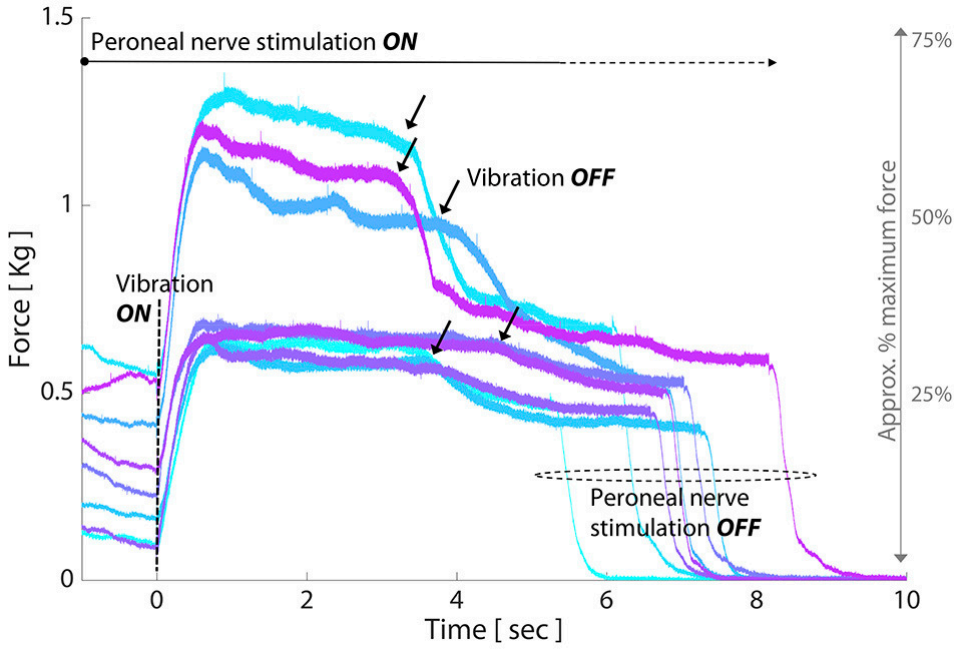
TVR responses in decerebrate cat follow similar trends to paretic elbow flexors post-stroke as preactivation increases. Each colored line represents the force evoked in response to a single TVR trial at a given level of preactivation. Crossed extension reflex is initiated prior to vibration onset (*t* = 0 s) to achieve a stable preactivation force and is maintained throughout the duration of vibration. *Y-axis*: evoked force (Kg); *x-axis*: time (sec); black arrows: vibration offset. TVR-evoked force amplitude exhibits negligible change with increased preactivation, and involuntary force production is maintained after cessation of vibration (black arrows) and until peroneal nerve stimulation is stopped.

We found that the pattern of TVR-evoked force in the decerebrate cat was strikingly similar to that of the paretic elbow flexors in individuals with chronic hemiparetic stroke (Figure 5). Specifically, we found that increasing preactivation force was *not* associated with a larger TVR-evoked force amplitude, with virtually no correlation between the two variables (*r* = −0.10; *p* = 0.8). In comparison, there was a higher correlation between preactivation force and the rising slope of the TVR-evoked force (*r* = 0.48) and time to 85% max TVR-evoked force (*r* = −0.62); however, neither of these correlations reached significance at the *p* < 0.05 level with the amount of data available (*p* = 0.19 and *p* = 0.08).

**Figure 6:**
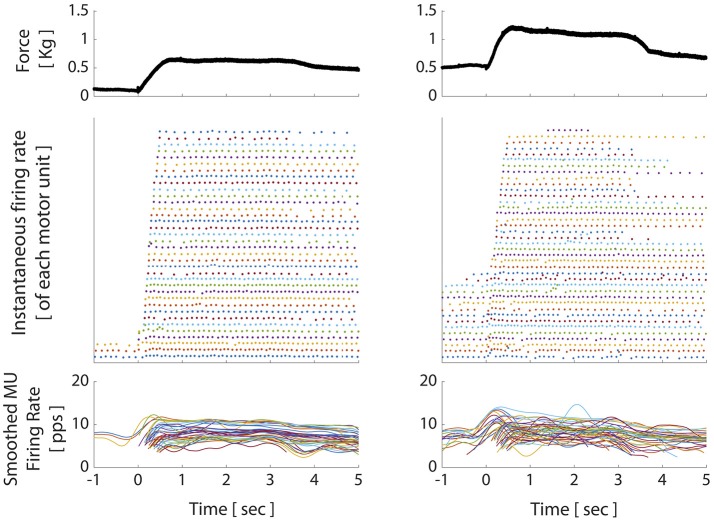
Motor unit firing patterns in decerebrate cat during crossed extension reflex and TVR trials. Top panel: muscle force; middle panel: instantaneous firing rate individual motor units recruited during the corresponding force trace (each color/row represents a separate motor unit); bottom panel: smoothed motor unit firing rates during the corresponding force trace. X-axis for all panels: elapsed time, relative to onset of vibration. Increasing preactivation force (mediated by the crossed extension reflex) led to increasing motor unit recruitment, as seen in left and right middle panels from *t* = −1:0 s. Vibration onset was marked primarily by further increases in motor unit recruitment and less so by increases in firing rate of previously recruited units. After vibration stops (at *t* = 3.5 s), persistent motor unit firing is evident, particularly in low-threshold units; a hallmark of motoneuron PICs.

To further investigate how the TVR-evoked torque arose, we utilized motor unit spike trains to characterize motor unit recruitment and rate modulation. Motor unit spike trains for two TVR trials with different preactivation levels are represented in Figure 6 by their instantaneous firing rates. Thirty-six motor units were decomposed from the trial with lower preactivation (left panel) and 39 motor units were decomposed from the trial with higher preactivation (right panel). Qualitatively, motor units followed expected trends of recruitment order and rate modulation during preactivation and vibration: increasing preactivation increased the number of motor units recruited before vibration, and vibration led both to recruitment of additional motor units and an increased firing rate in motor units previously recruited with preactivation (Figure 6). However, it can be seen that for both levels of preactivation, recruitment of additional motor units is the dominant contributor to the development of TVR-evoked force, with an increased firing rate of previously recruited motor units contributing to a lesser extent.

The decerebrate cat preparation also allowed us to examine potential post-vibration force production (given the lack of descending voluntary drive as a confounding factor), and, by extension, the ability of a purely spinal mechanism to account for any such effects. When vibration was discontinued, force persisted at an elevated level until peroneal nerve stimulation was discontinued (Figures 5, 6; force produced after *t* = ~3.5–4.5 s). This persistent elevated force was accompanied by sustained firing of the vibration-recruited motor units despite removal of the vibration stimulus. Interestingly, we also observed differences between the two trials in the proportion of vibration-recruited motor units that demonstrated sustained firing. For the trial with lower preactivation, 32 of the 33 vibration-recruited motor units demonstrated sustained firing. For the trial with higher preactivation, however, only 12 of the 24 vibration-recruited motor units demonstrated sustained firing through the end of the trial.

## Discussion

We demonstrated that TVR-evoked torque amplitude in the paretic arm of individuals with chronic hemiparetic stroke significantly exceeds that of the non-paretic arm when muscles are preactivated to the same degree. Interestingly, within both the paretic and the non-paretic arm, TVR-evoked torque amplitude did not increase further when preactivation increased from 5 to 15% MVT. The rising *slope* of TVR-evoked torque did, however, increase with increasing preactivation in the paretic arm, whereas non-paretic values were not influenced by preactivation. Likewise, parameters extracted from the nonlinear model of TVR-evoked torque changed as a function of preactivation level only in the paretic arm. These findings contrast with the results of many classical stretch reflex paradigms, which suggest that reflex amplitude should equilibrate between paretic and non-paretic muscles when preactivation is matched (Lee et al., 1987; Burne et al., 2005; McPherson et al., 2017). Below, we discuss potential neural mechanisms that could underlie our findings.

### Increased TVR-evoked torque amplitude in the paretic vs. non-paretic limb

One potential explanation for the increased amplitude of TVR-evoked torque in the paretic compared to the non-paretic arm (Figure 1) could be an increased monoaminergic drive to spinal motoneurons from the brainstem ponto-medullary reticular formation (PMRF). The PMRF has both a descending motor and a descending neuromodulatory component (Holstege and Kuypers, 1987), and recent evidence suggests that the motor component is progressively recruited post-stroke as volitional force production increases in the paretic arm (McPherson et al., 2018). The neuromodulatory component uses the monoamines serotonin and norepinephrine to regulate spinal excitability (Holstege and Kuypers, 1987; Hochman et al., 2001; Heckman et al., 2003), and is co-activated with the motor component (Veasey et al., 1995; Jacobs et al., 2002; Chan et al., 2006; Schwarz et al., 2008). Monoamines are uniquely excitatory to motoneurons, for example amplifying post-synaptic potentials 3–5-fold via PICs (Schwindt and Crill, 1977; Perrier and Hounsgaard, 2003; Heckman et al., 2009) while depolarizing the resting membrane potential and hyperpolarizing the spike threshold (Fedirchuk and Dai, 2004; Harvey et al., 2006). Thus, an increased descending monoaminergic drive could result in elevated TVR responses in paretic muscles through greater PIC-mediated amplification of motoneurons and/or by additional motor unit recruitment during vibration. It should be noted, however, that previous investigations have yet to find changes in PIC amplitude in paretic muscles relative to non-paretic muscles post-stroke (Mottram et al., 2009, 2010), although methodological differences prevent a direct comparison to the findings presented here. Also, it should be noted for clarification that a *decrease* in monoaminergic drive may contribute to hyperreflexia after spinal cord injury (as opposed to the hypothetical increase described here post-stroke), because a lack of monoamines below the lesion appears to drive adaptive processes that result in constitutive activity of monoaminergic receptors on motoneurons (Murray et al., 2010).

Alternatively, it is possible that a tonic excitatory ionotropic drive to paretic motor pools could have contributed to the increased TVR amplitude we observed in the paretic arm. Such a drive could arise from ionotropic reticulospinal or vestibulospinal pathways following stroke-induced damage to primary motor resources (Mottram et al., 2009, 2010; Miller and Dewald, 2012; Miller D. M. et al., 2014; McMorland et al., 2015; McPherson et al., 2017), and would provide a maintained depolarizing input to motor pools innervating the paretic muscles. This would bring motoneurons closer to firing threshold, enabling vibration to more readily recruit them. It could also depolarize a small portion of the motor pool above firing threshold, leading to spontaneous motoneuron firing (Mottram et al., 2010)—a common finding post-stroke.

Our findings contrast with some aspects of the ionotropic drive hypothesis, particularly as they relate to other studies that cite this mechanism as possible explanation for exaggerated stretch-sensitive reflexes post-stroke. For example, when stretch reflexes are evoked by imposed joint excursions in the paretic and non-paretic arms at matched levels of preactivation, the difference in reflex response between arms is often extinguished (Lee et al., 1987; Burne et al., 2005; McPherson et al., 2017). Results from these studies suggest that the postulated tonic ionotropic drive is low enough that even small amounts of preactivation normalize the difference in resting excitability attributed to the tonic drive. Although a key assumption of the tonic ionotropic drive hypothesis is that preactivation is an effective means of balancing the excitability offset between limbs, it remains unknown if this is indeed the case. Nevertheless, if preactivation *does* abolish the resting excitability imbalance between limbs, then our results cannot be explained solely by a tonic ionotropic drive to motor pools innervating the paretic muscles. If that were the case, then TVR responses in paretic and non-paretic limbs would equilibrate with preactivation.

Changes in spinal circuits post-stroke could have also contributed to the increased TVR amplitude we observed in the paretic arm, although our experimental paradigm and analyses were not able to directly measure or clearly infer the potential contribution of such changes to our results. Nevertheless, there is evidence that the net effect of Group Ib afferent feedback transitions to from a combination of inhibition and excitation (Houk et al., 1970; Conway et al., 1987; Pearson and Collins, 1993; Prochazka et al., 1997) to preferential excitation in motor pools innervating the paretic muscles post-stroke (Delwaide and Oliver, 1988). While tendon vibration provides a strong volley of a Group Ia afferent feedback from muscle spindles, it also provides afferent information from Golgi tendon organs via Group Ib fibers. Given that Golgi tendon organs/Group Ib fibers are progressively activated by vibration as background muscle tension increases (Fallon and Macefield, 2007), a lateralized shift in Ib feedback toward excitation could explain a portion of our elevated paretic limb TVR responses. Further, if descending monoaminergic drive is indeed increased, interneuron responses to Group Ia and Ib feedback could be potentiated (Jankowska et al., 2000). And, while the effects of monoamines on Group II-evoked responses are both complex and incompletely characterized (Jankowska et al., 2000; Grey et al., 2001; Kurtzer et al., 2018), changes in this feedback mechanism would likely alter TVR responses as well. Likewise, a reduction of presynaptic inhibition, which is associated with spasticity following multiple sclerosis, spinal cord injury, and possibly stroke (Faist et al., 1994; Aymard et al., 2000; Morita et al., 2001; Nielsen et al., 2005; Lamy et al., 2009), could differentially alter reflex gain between arms and contribute to elevated TVR responses in the paretic arm.

### Invariance of TVR-evoked torque amplitude to increasing preactivation

It is unclear why the magnitude of TVR-evoked torque did not increase in either limb when preactivation increased from 5 to 15% MVT. Indeed, it is well accepted that the amplitude of stretch reflexes elicited by imposed joint excursions increases with increasing preactivation in paretic, non-paretic, and control limbs. Further, Henneman's size principle (Binder et al., 1983; Bawa et al., 1984; Henneman, 1985) suggests that, at low force levels, recruitment of progressively larger force motor units will impart an upwards curvature to the input-output function, regardless of the motoneuron input mixture (Heckman, 1994). This should cause TVR-evoked torque amplitude to increase with pre-activation level in both arms.

Two potential mechanisms could contribute to this unexpected finding. In the first, synaptic inhibition could simultaneously increase and decrease in direct proportion to preactivation level, a phenomenon known as “proportional,” or “balanced,” inhibition (Powers et al., 2012; Powers and Heckman, 2017). Because monoaminergic actions on motoneurons are exquisitely sensitive to synaptic inhibition (Johnson and Heckman, 2014), even small amounts of additional inhibition during contraction could prevent the predicted increase in TVR response amplitude. In the second scenario, monoaminergic drive to the paretic limb could be elevated yet relatively static post-stroke, contributing approximately the same net amount of excitation across the preactivation levels tested here. Because the non-paretic limb is presumably less reliant on monoaminergic drive for motoneuron activation than the paretic limb (due to an intact corticospinal tract), it is more likely to maintain an appropriate level of baseline inhibition, retain the capacity for presynaptic and reciprocal inhibition, and use a homeostatic “push-pull” method to regulate the excitation/inhibition balance during contraction (Powers et al., 2012; Powers and Heckman, 2017). These characteristics would enable more precise control of synaptic integration, potentially leading to similar levels of motoneuron recruitment during vibration for the relatively low submaximal contractions investigated here.

### Corroboration with animal model

To further explore our finding that paretic limb TVR-evoked torque amplitude was not different between 5 and 15% preactivation levels post-stroke, we investigated the impact of increasing preactivation level on the amplitude and rate of rise of TVR-evoked force in a decerebrate cat model. This model has a strong descending monoaminergic drive, the magnitude of which is not modified by vibration or crossed-extension reflex inputs (Lee and Heckman, 1996; Hyngstrom et al., 2008). Thus, force output in this model is due only to three factors: the sustained monoaminergic drive, Ia input (via vibration), and the resulting input-output function of the motoneurons. Like our human-subjects findings, the magnitude of TVR-evoked force in the cat did not scale with preactivation level. Also similar to our human-subjects findings, there was a stronger correlation between preactivation level and rate of rising force; however, the correlation was not statistically significant in cat data. Also, because vibration is very selective for Ia inputs in the decerebrate cat model (Hyngstrom et al., 2008), the similarity of our cat and human-subjects results suggests that monosynaptic Ia feedback was the predominant driver of motoneuron activation in the human-subjects torque as well, with less influence from Ib or other sensory inputs.

Importantly, the decerebrate cat model also enabled us to investigate force production *after* vibration was discontinued, which was not possible in our human-subjects experiments. We found maintained, elevated force post-vibration rather than a return to preactivation level or the relaxed state. This behavior is characteristic of PICs in motoneurons (Heckman et al., 2008) and is consistent with post-vibration TVR responses seen in humans when no preactivation is present, as was detailed in our previous study (McPherson et al., 2008). The finding that sustained firing of vibration-recruited motor units accompanied the post-vibration force maintenance is highly consistent with activation of PICs, which characteristically enable a motoneuron to continue firing after removal of the excitatory stimulus that recruited it. Further, our observation that a greater percentage of motor units exhibited sustained firing in the trial with lower preactivation (32 of 33) compared to the trial with higher preactivation (12 of 24) is consistent with the greater impact that PICs have on sustained firing in low vs. higher threshold motor units (Heckman et al., 2008).

Together, these findings corroborate and extend key aspects of our overall unexpected human-subjects findings, and reinforce the notion that monoaminergic mechanisms are a potential component of the observed paretic arm TVR responses. However, it should be noted that in the decerebrate cat there is no natural analog to the non-paretic arm in humans, and it is unknown how the afferent drive provided by peripheral nerve stimulation in the cat compares to volitional preactivation in humans with regard to its inhibitory and excitatory content.

### Slope of TVR-evoked torque and modeled torque gain

We found that the slope of TVR-evoked torque amplitude and the gain of the modeled torque both increased with increasing preactivation in the paretic limb. These findings are consistent with an increased influence of monoamines in paretic motor pools, which could result in a combination of larger PICs in recruited motoneurons, more motoneurons recruited, and/or a reduced time to PIC activation as preactivation increases.

PIC activation time is proportional to the difference in membrane potential and PIC threshold potential (Heckman and Lee, 2001). Thus, as membrane potential depolarizes toward PIC threshold, the time required to activate the PIC by a given stimulus is reduced. Here, this phenomenon could occur when transitioning from the relaxed case to 5 and 15% MVT preactivation: as preactivation increases, an increasing amount of motoneurons are brought near to or above their PIC threshold. Assuming that monoaminergic drive would be elevated to paretic motoneurons compared to non-paretic motoneurons, PICs will be more rapidly activated in paretic motoneurons when vibration begins. The net effect of an increased PIC activation rate will be a faster rate of force production, and thus a higher TVR slope and gain. By comparison, the lower slope and modeled torque gain in the non-paretic limb could reflect a lack of robust neuromodulatory effects.

### Comparison with stretch reflex studies

Finally, an intriguing question raised by these results is why TVR responses continue to be elevated in the paretic limb compared to the non-paretic limb at matched levels of preactivation, yet stretch reflex responses equilibrate under analogous conditions. It is conceivable that this distinction is related to the potency of vibration as an input to motor pools compared to imposed joint excursions. Indeed, it has been suggested that vibration elicits more robust activation of Ia afferents than group Ib or II fibers, which may be activated more strongly by joint excursion (Matthews, 1984). More specifically, if vibration elicits a larger response in spinal motor pools than joint excursion, then lingering imbalances in motor pool excitability that were not well controlled by preactivation could “re-appear” through enhanced motor unit recruitment during vibration. Or, put another way, if joint excursion is a relatively modest stimulus, it may not recruit substantially more motor units over those already recruited via preactivation. This could mask persistent excitability imbalances.

This explanation seems unlikely, however, both because spindle-mediated feedback appears to be unchanged post-stroke (Hagbarth et al., 1973) and because our TVR paradigm actually appears to evoke a relatively small motor output compared to the joint excursions typically used to gauge motor excitability. Indeed, we found that when preactivated to 5% MVT, vibration only increased net elbow flexion torque output to ~13% MVT in the paretic limb. By comparison, a rapid joint excursion (270°/s) at 5% MVT preactivation can routinely drive reflex responses that exceed 20–25% MVT (McPherson et al., 2017), even though the stretch duration is only ~300 ms (compared to 5 s for vibration). Further, if our paretic limb TVR responses are compared to previously reported stretch reflex data post-stroke (McPherson et al., 2017), we find that they most closely match an approximately quasi-static imposed joint excursion (6°/s) in terms of rise time and amplitude. Thus, it remains an open question as to why the two stimuli modalities yield such differing responses.

### Limitations

Our study has important limitations. First, we reiterate that because our human-subjects experiments used indirect probes of spinal neural excitability, our interpretation of the underlying mechanisms remains inferential. Additionally, we did not conduct TVR analyses in individuals without neurological injury. Although this choice was motivated by an attempt to limit variability, the ipsilesional arm is not truly unaffected post-stroke, and thus, future studies are warranted. We also did not acquire elbow flexion/extension torque after cessation of vibration in the human-subjects portion of the study. While this choice was made to avoid the confound of volitional activity in any persistent muscle activation post-TVR, it prevents our study from assessing the impact of preactivation on persistent motoneuron firing—an important component of PICs. And finally, regarding the decerebrate cat experiments: because the primary purpose of this preliminary cat model was to provide context for the mechanical response to vibration in a situation with known elevation of monoaminergic drive, we did not design the protocol with the specific goal of acquiring EMG data amenable to studying PICs. As a result, we are not able to make inferences about PICs from these data.

## Conclusions

Our results suggest that a combination of both ionotropic and neuromodulatory mechanisms likely underlie the exaggerated TVR responses we observed in the paretic elbow flexors post-stroke, although the relative impact of each remains unclear. The finding that TVR-evoked torque amplitude did not change with preactivation level could imply a more pronounced role for synaptic inhibition in motor pools innervating the paretic muscles than previously thought. For example, an increase in inhibitory drive as volitional motor output increases (Powers and Heckman, 2017; Revill and Fuglevand, 2017) would reduce PIC-mediated effects on motoneuron firing dynamics, potentially stabilizing TVR-evoked torque output. Advanced yet available technologies such as high-density surface EMG grids and motor unit decomposition algorithms, which can be incorporated into both human experiments and animal preparations (Miller L. C. et al., 2014; McPherson et al., 2016; Johnson et al., 2017), will be essential for more fully characterizing these effects.

## Author contributions

All authors contributed to the conception and design of the work; JM, LM, CT, ME contributed to the acquisition of data; JM, LM, CT contributed to analysis of data; all authors contributed to the interpretation of data and drafting, revising and approving the manuscript for publication. All authors are in agreement to be accountable for all aspects of the work.

### Conflict of interest statement

The authors declare that the research was conducted in the absence of any commercial or financial relationships that could be construed as a potential conflict of interest.
